# Domains of transmission and association of community, school, and household sanitation with soil-transmitted helminth infections among children in coastal Kenya

**DOI:** 10.1371/journal.pntd.0007488

**Published:** 2019-11-25

**Authors:** William E. Oswald, Katherine E. Halliday, Carlos Mcharo, Stefan Witek-McManus, Stella Kepha, Paul M. Gichuki, Jorge Cano, Karla Diaz-Ordaz, Elizabeth Allen, Charles S. Mwandawiro, Roy M. Anderson, Simon J. Brooker, Rachel L. Pullan, Sammy M. Njenga

**Affiliations:** 1 Faculty of Infectious and Tropical Diseases, London School of Hygiene & Tropical Medicine, London, United Kingdom; 2 Eastern and Southern Africa Centre of International Parasite Control, Kenya Medical Research Institute, Nairobi, Kenya; 3 Pwani University Bioscience Research Centre, Pwani University, Kilifi, Kenya; 4 Faculty of Epidemiology and Population Health, London School of Hygiene & Tropical Medicine, London, United Kingdom; 5 Faculty of Medicine, Department of Infectious Disease Epidemiology, London Centre for Neglected Tropical Disease Research, School of Public Health, St Mary's Campus, Imperial College London, London, United Kingdom; Institute for Disease Modeling, UNITED STATES

## Abstract

**Introduction:**

Few studies have simultaneously examined the role of sanitation conditions at the home, school, and community on soil-transmitted helminth (STH) infection. We examined the contribution of each domain that children inhabit (home, village, and school) to STH infection and estimated the association of STH infection with sanitation in each domain.

**Methods:**

Using data from 4,104 children from Kwale County, Kenya, who reported attending school, we used logistic regression models with cross-classified random effects to calculate measures of general contextual effects and estimate associations of village sanitation coverage (percentage of households with reported access to sanitation), school sanitation coverage (number of usable toilets per enrolled pupil), and sanitation access at home with STH infection.

**Findings:**

We found reported use of a sanitation facility by households was associated with reduced prevalence of hookworm infection but not with reduced prevalence of *T*. *trichiura* infection. School sanitation coverage > 3 toilets per 100 pupils was associated with lower prevalence of hookworm infection. School sanitation was not associated with *T*. *trichiura* infection. Village sanitation coverage > 81% was associated with reduced prevalence of *T*. *trichiura* infection, but no protective association was detected for hookworm infection. General contextual effects represented by residual heterogeneity between village and school domains had comparable impact upon likelihood of hookworm and *T*. *trichiura* infection as sanitation coverage in either of these domains.

**Conclusion:**

Findings support the importance of providing good sanitation facilities to support mass drug administration in reducing the burden of STH infection in children.

## Author summary

Infection by the soil-transmitted helminths (STH) whipworm and hookworm results from either ingestion of eggs or larvae or through skin exposure to larvae. These eggs and larvae develop in suitable soils contaminated with openly-deposited human faeces. Safe disposal of faeces should reduce transmission of STH, yet evidence of the impact of sanitation on STH transmission remains limited. A large, community-wide survey was conducted in 2015 to measure prevalence of STH infections in Kwale County, Kenya. Here, we used this data and observations made in schools to examine the relationship between sanitation conditions at home, school, and village and the presence of STH infection among 4,104 children who attend school. We found that sanitation access at home and school sanitation coverage (number of usable toilets per enrolled pupil), but not the overall level of village sanitation coverage (percentage of households with reported access to sanitation), was protective against hookworm infection. In contrast, only high village sanitation coverage, but not home or school sanitation, was protective against whipworm infection. Current STH control strategies emphasise periodic deworming of at-risk populations, including school-age children. Our findings highlight the need for continued efforts, alongside deworming, to extend access to good sanitation facilities at homes, schools, and across communities.

## Introduction

In 2016, it was estimated that world-wide more than 1.5 billion people were infected with at least one species of soil-transmitted helminth (STH)[1]. Infection by roundworm, *Ascaris lumbricoides*, and whipworm, *Trichuris trichiura*, results from ingestion of embryonated eggs. The hookworms, *Necator americanus* and *Ancylostoma duodenale*, infect humans through skin exposure to or ingestion of larvae (*A*. *duodenale)* that develop in warm, moist soils from eggs in openly-deposited human faeces [2, 3]. Preventing human contact with excreta through consistent safe disposal of faeces should reduce STH transmission, yet evidence of the impact of sanitation on STH remains limited [4–6]. The concept of private and public domains of transmission for STH has been described previously [7, 8], but to our knowledge few studies have simultaneously examined the role of sanitation conditions at the home, school, and community on STH infection [9–11].

Multilevel statistical models provide a means to estimate effects of individual factors, using measures of association. They can simultaneously assess general contextual effects upon individual health outcomes, using measures of within-unit clustering and between-unit heterogeneity [12, 13]. Such models provide a useful complement to mathematical modelling of transmission dynamics for examining specific effects and possible areas for intervention [14]. Identifying effects of sanitation within each of the domains that a child inhabits will contribute to evidence for the prioritization of sanitation promotion activities alongside the regular deworming of at-risk populations, including school-aged children, recommended by the World Health Organization [15, 16].

Employing multilevel modelling, we investigate the relative importance of household, village, and school domains and estimate the association of sanitation in each domain on STH infection among Kenyan children who attend school.

## Methods

### Study population

The study took place in Kwale County on the south Kenyan coast. Data were collected during a cross-sectional parasitological survey conducted between March-May 2015 as the baseline for TUMIKIA, a randomised, controlled trial to evaluate the impact and cost-effectiveness of school-based versus community-wide deworming on STH transmission (NCT02397772, www.clinicaltrials.gov).

Study design, baseline findings, and impact have been described previously [17–19]. The TUMIKIA trial was implemented via community units (CUs)—government health-service delivery structures of approximately 1,000 households, comprising 2 to 7 villages. For the baseline survey, 225 households were randomly selected within 120 CUs. Among consenting households, a structured questionnaire was conducted with the head of household or primary caregiver to collect information on demographics, ownership of key assets, and sanitation, hygiene, and water conditions [20]. One household member (aged ≥ 2 years) was randomly selected to provide a stool sample. A questionnaire was then conducted with individuals who provided samples or their caregiver (for those under 5 years old) to collect information on deworming within the last year and observe their footwear. School facility surveys were conducted across Kwale County in June 2015 and July 2016. During visits, student enrolment was recorded from school registers, and sanitation conditions were assessed using structured observations. One school surveyed in 2015 was missing the total number of children enrolled, so the 2016 enrolment number was used. For 51 schools attended by children for which we had no 2015 data, enrolment and sanitation conditions from the 2016 survey were used. All data were collected on smartphones running the Android operating system (Google, Mountain View, CA, USA) using SurveyCTO (Dobility, Inc., Cambridge, MA, USA). Records from school and household surveys were linked based on the school each child reported attending. Geographic coordinates (based on WGS84 datum) were systematically collected at each household and school using the smartphones’ global positioning systems. Missing coordinates for 4 schools were obtained from Google Maps (Google, Mountain View, CA, USA). Children were excluded *a priori* if they lived in villages or attended schools in semi-arid areas unsuitable for STH transmission [19]. Children were eligible if they were sampled in the 2015 TUMIKIA baseline parasitological survey, aged 5 to 14 years, and reported attending school.

### STH infection

Kato-Katz microscopy was used to enumerate STH eggs (*A*. *lumbricoides*, *T*. *trichiura*, and hookworm) per gram of stool. Duplicate slides were prepared from a single stool sample and read by independent microscopists. STH infection was classified based on categories of infection intensity [21], and frequencies were tabulated across categories of household, community, and school sanitation as detailed below. For both hookworm and *T*. *trichiura*, our outcome was a dichotomous indicator for the presence of > 0 eggs in stool samples (i.e. prevalence). *A*. *lumbricoides* was not examined in detail since few cases were detected.

### Sanitation measures

The measure for household sanitation access was combined from reported use of a toilet facility on or off the household’s compound. Using all households sampled per village for the TUMIKIA baseline survey, this measure of household sanitation was aggregated for village sanitation coverage, as the percentage of households with reported access to sanitation. During structured observations at schools, the number of latrines considered usable (not assigned to teachers, locked, or with full pits) was quantified, excluding urinals, for both girls and boys. School sanitation coverage was calculated as the number of usable toilets per enrolled pupil, in contrast to the indicator of students per toilet [22]. Village and school sanitation coverage were categorised based on estimated quartiles to explore possible non-linear relationships during modelling.

### Covariates

Candidate individual covariates included median-centred age in years, sex, a dichotomous indicator for observed shoe wearing, an indicator for whether household floor was covered (relative to an earth/sand floor), and an indicator for reported deworming with albendazole in past year. Relative wealth was determined using a factor analysis, separately for rural and urban households, of dichotomous indicators for ownership of household assets, household wall and roof materials, and household access to electricity. The indices were then divided into quintiles within urban and rural settings, and then second, third, and fourth quintiles were combined prior to analysis [19]. For villages and schools, environmental and sociodemographic conditions were hypothesized to influence both STH transmission and sanitation coverage. Areas were classified as urban, peri-urban, or rural using 2015 estimates of population density [23]. Percentage soil sand content was extracted from soilgrids.org at a resolution of 250 m [24]. Sand content was first categorised into tertiles and then a dichotomous indicator for highest sand content. A measure of aridity was obtained from the Consortium for Spatial Information (CGIAR-CSI) [25]. These measures were assembled in a geographic information system using ArcGIS 10.3 (ESRI, Redlands, CA, USA), and values were extracted for each school and household based on GPS coordinates [19]. We then aggregated mean continuous and mode categorical household values per village, using all households sampled per village.

### Ethical approval

The TUMIKIA trial protocol was approved by the Kenya Medical Research Institute and National Ethics Review Committee (SSC Number 2826) and the London School of Hygiene & Tropical Medicine (LSHTM) Ethics Committee (7177). Written informed consent was sought from the household head or adult answering the household-level questionnaire. Written informed consent was also sought from adults (≥18 years) selected to provide the stool sample and complete the individual-level questionnaire. Parental consent was sought for individuals aged 2 to 17 years, and written assent was additionally obtained from children aged 13 to 17 years. All information and consent procedures were conducted in Kiswahili.

### Statistical analyses

We estimated associations between sanitation conditions in the domains of interest (household, school, village) and presence of hookworm and *T*. *trichiura* infection, separately, using logistic regression models with cross-classified (non-nested) random effects to account for membership of children within village of residence and school attended.

We fit a series of generalised mixed models (with logit link), excluding observations with missing outcome or covariate information, which assumes the probability of having complete data is independent of the outcome after adjusting for included covariates. First, we fit an intercept-only model containing school- and village-specific random effects to quantify between school and between village variation in STH infection. We then fit a series of models to explore changes in general contextual effects for village and school domains with the inclusion of fixed sanitation effects and confounders at the different levels. Next, we fit models with fixed effects for sanitation conditions in each domain separately and then together in a combined crude model. Finally, we fit a model containing all sanitation effects, adjusting for potential confounders. Confounders were selected from candidate individual, village, and school covariates based on existing knowledge and encoding possible causal relationships in directed acyclic graphs. We then implemented d-separation in DAGitty to identify minimal sufficient sets of available covariates to control to estimate effects of sanitation on both hookworm (S1 Fig and S1 Text) and *T*. *trichiura* infections (S2 Fig and S2 Text) [26, 27]. Estimation used Hamiltonian Monte Carlo sampling to improve mixing and reduce auto-correlation [28]. We conducted a sensitivity analysis excluding outliers in and missingness of distance from child’s home to reported school attended.

We report prevalence odds ratios (PORs) with 95% credible intervals (CIs) as fixed effects for sanitation in each domain. In addition to the specific contextual (fixed) effects for village and school sanitation, we also calculated measures to quantify general contextual effects on individual infection [13, 29]. Proportion of total individual variation in the outcome attributable to between-school and between-village variation, or Variance Partition Coefficient (VPC), was calculated using the latent variable method. This method converts individual variance to the logistic scale from the probability scale and assumes an underlying continuous propensity for infection following a logistic distribution with individual variance of π^2^/3 [30]. The VPC is interpreted in the same way as the Intraclass Correlation Coefficient (ICC) only when the structure of the information is hierarchical. We calculated median odds ratios (MORs), as measures of residual heterogeneity on the odd ratio scale. MORs are always greater than or equal to 1 (MOR = 1 if there is no variation between areas) and interpreted as the median value of the odds ratios between comparable individuals drawn randomly from high and low risk areas and always having the individual from the higher risk area in the numerator. MORs are useful as they measure how much individual infection is determined by domain membership and are directly comparable to fixed effects [30]. The VPC is recommended, however, for estimating general contextual effects as a measure of clustering that incorporates both between- and within-area variance [12]. We calculated 80% interval odds ratios (80% IORs) for village and school sanitation fixed effects. This measure does not reflect the estimate’s precision but instead is recommended to consider residual variation in the interpretation of fixed effects [31]. A wider interval indicates greater unexplained between-area variation, and the inclusion of 1 indicates between-area variance is large compared to the specific fixed effect. Analyses were conducted in Stata 15 (StataCorp LP, College Station, TX, USA) and in R 3.5 (r-project.org) using the ‘brms’ package.

## Results

**Fig 1:**
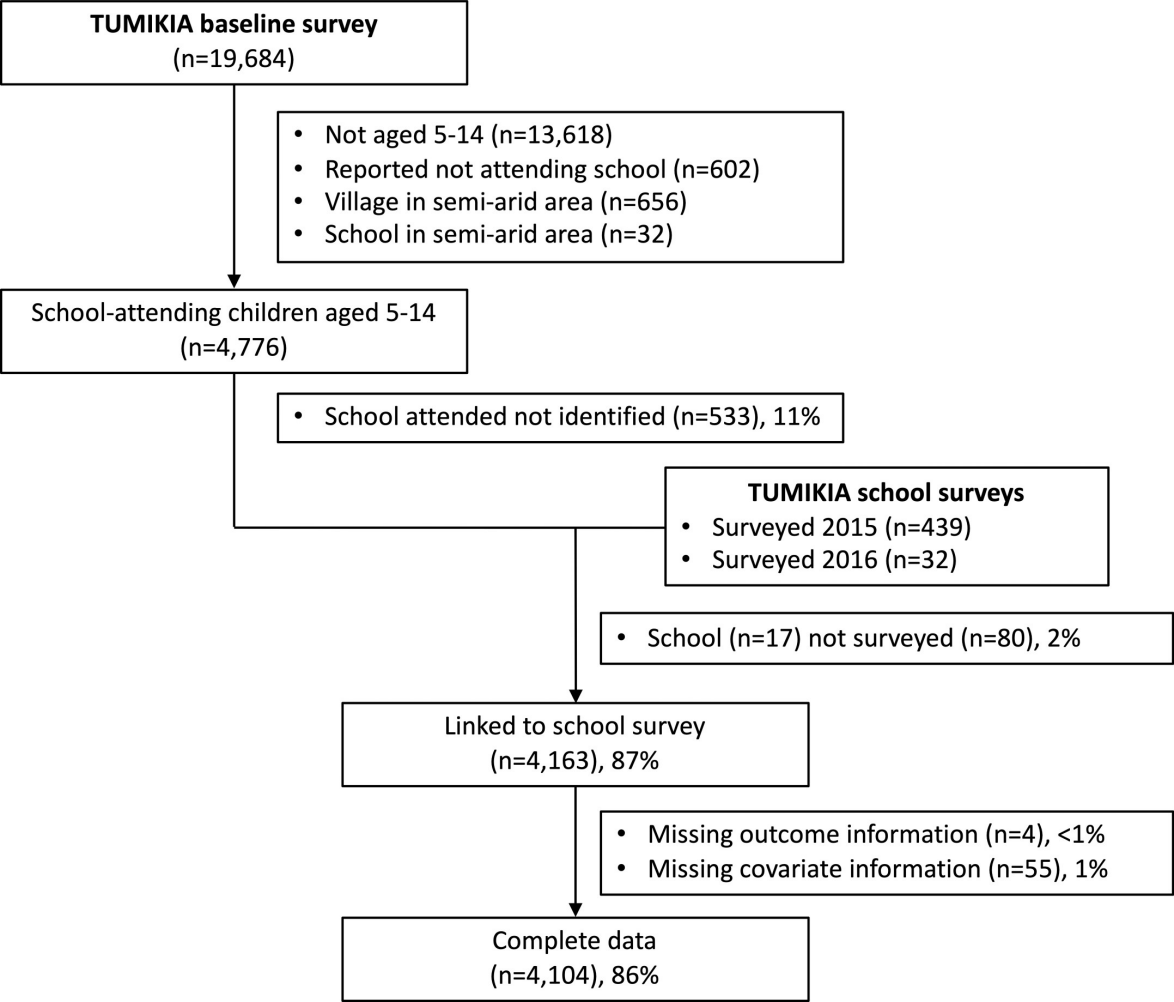
Flow chart of participants in the TUMIKIA baseline survey who were included in the current analysis. Eligible sample included children aged 5–14 years who reported attending school and not residing in villages or attending schools in semi-arid areas. The proportion of eligible subjects with complete data is 86% (4,104/4,776).

Of 6,066 school-aged children with matched samples in the survey, 602 (10%) reported not attending school, and 688 (11%) reported attending school but resided in a village or were linked to a school in a semi-arid area (Fig 1). Among these excluded children, prevalence of infection was 3% (22/688), < 1% (4/688), and 0% (0/688) for hookworm, *T*. *trichiura* and *A*. *lumbricoides*, respectively. Of 4,776 eligible children, the school reported to be attended was identified for 4,243 children (89%) and school survey data was available for 4,163 children (87%). Eligible children without school information were younger, more often being boys, less likely to have been dewormed, and less poor (S2 Table). Data were available for 4,104 eligible children (86%).

**Fig 2:**
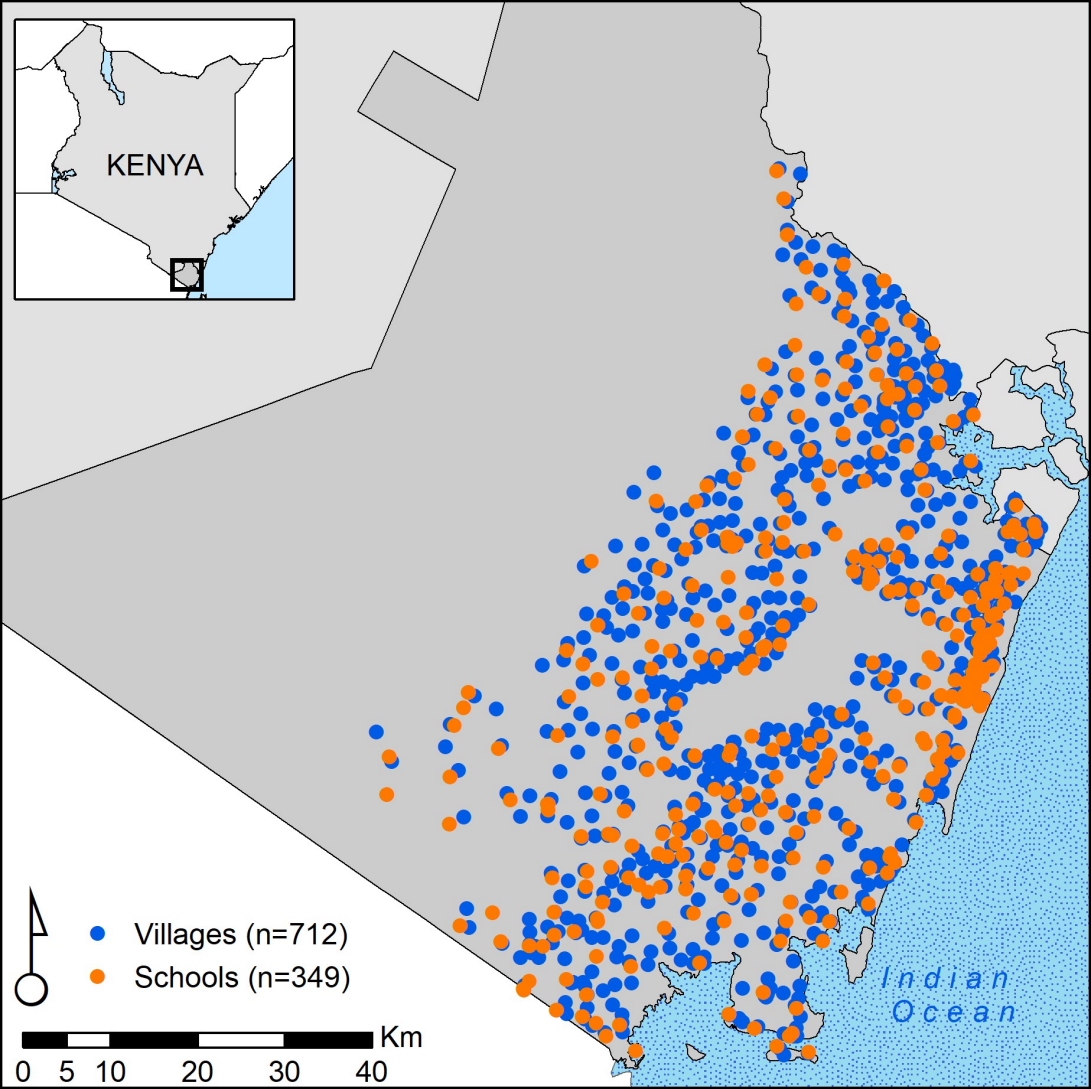
Village of residence and attended schools among 4,104 school-attending children aged 5–14 years in Kwale County, Kenya, 2015.

**Fig 3:**
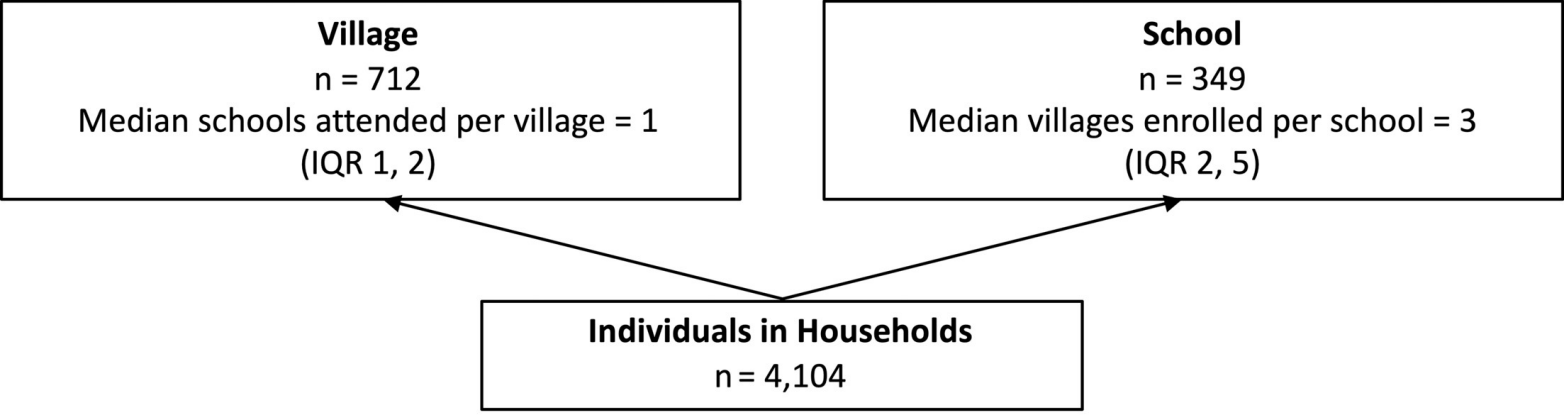
Diagram for the classification model of individuals, villages, and schools.

Participants resided in 712 villages (median sampled per village 4, range 1, 38) and reported attending 349 schools (median sampled per school 9, Range 1, 49) (Fig 2). Fig 3 describes the structure of the data. In half of all villages, resident children reported attending the same school (range 1 to 8 schools per village). Included schools enrolled children from 1 up to 13 villages. Table 1 describes individual, household, and village characteristics of included children.

**Table 1:** Summary characteristics for 4,104 school-attending children in coastal Kenya, 2015.

Characteristics		No. or Mean	% or SD
**Individual level (n = 4,104)**			
	Age (years)	9.46	2.65
	Being girls	2,129	51.88
	Observed wearing shoes	1,443	35.16
	Reported deworming in past year	2,219	54.07
	Reported household access to toilet	2,255	54.95
	Reported improved water source	2,216	54.00
	Missing	13	0.32
	Time to fetch water, < 30 min	3,344	81.48
	Missing	14	0.34
	Covered floor	833	20.30
	Household wealth quintile		
	Most poor, quintile 1	1,190	29.00
	Quintiles 2–4	2,158	52.58
	Least poor, quintile 5	756	18.42
	Distance to school (km) (n = 3,906)	2.25	5.82
**Village level (n = 712)**			
	Proportion reporting toilet access	0.53	0.31
	Urbanization		
	Rural	545	76.54
	Periurban	141	19.80
	Urban	26	3.65
	Aridity index		
	Dry sub-humid (> 0.5–0.65)	284	39.89
	Humid (> 0.65)	428	60.11
	Soil sand content ≥ 62%	256	35.96
**School level (n = 349)**			
	Usable toilets per pupil	0.03	0.02
	Pupils per usable toilet	57.79	47.35
	Total enrolment	479.47	270.67
	Urbanization		
	Rural	234	67.05
	Periurban	84	24.07
	Urban	31	8.88
	Aridity index		
	Dry sub-humid (> 0.5–0.65)	130	37.25
	Humid (> 0.65)	219	62.75
	Soil sand content ≥ 62%	126	36.10

Overall prevalence of any detected infection was 17.8% and 6.0% for hookworm and *T*. *trichiura*, respectively, and most infections were classified as light (Table 2). Comparing MOR in Tables 3 and 4, school attended had greater impact than village of residence upon hookworm infection (School MOR 2.73, 95% CI 2.50, 2.98; Village MOR 2.09, 95% CI 1.74, 2.38). School and village membership had comparable impacts on *T*. *trichiura* infection (School MOR 2.92, 95% CI 2.40, 3.43; Village MOR 2.78, 95% CI 2.28, 3.29). Calculated VPC showed similar results.

**Table 2:** Intensity and any presence of STH infections by household, school, and village sanitation conditions among 4,104 school-attending children in coastal Kenya, 2015.

				Intensity			
Infection		N	None %	Light %	Moderate %	Heavy %	Any %
**Hookworm**	Overall	4,104	82.21	16.91	0.56	0.32	17.79
						
	Household sanitation access						
	Yes	2,255	84.12	15.25	0.40	0.22	15.88
	No	1,849	79.88	18.93	0.76	0.43	20.12
	School sanitation coverage (per 100)						
	< 1.49	1,182	81.30	17.77	0.68	0.25	18.70
	1.49–2.17	1,181	80.10	19.14	0.68	0.08	19.90
	2.18–3.13	1,077	83.84	15.51	0.46	0.19	16.16
	> 3.13	664	84.94	13.70	0.30	1.05	15.06
	Village sanitation coverage						
	≤ 0.25	792	83.59	15.53	0.63	0.25	16.41
	0.26–0.54	1,123	82.37	16.92	0.53	0.18	17.63
	0.54–0.81	1,130	77.88	20.71	0.88	0.53	22.12
	> 0.81	1,059	85.65	13.88	0.19	0.28	14.35
***T*. *trichiura***	Overall	4,104	94.05	5.43	0.49	0.02	5.95
						
	Household sanitation access						
	Yes	2,255	94.10	5.54	0.35	0.00	5.90
	No	1,849	94.00	5.30	0.65	0.05	6.00
	School sanitation coverage (per 100)						
	< 1.49	1,182	92.89	6.35	0.68	0.08	7.11
	1.49–2.17	1,181	95.17	4.40	0.42	0.00	4.83
	2.18–3.13	1,077	94.15	5.48	0.37	0.00	5.85
	> 3.13	664	93.98	5.57	0.45	0.00	6.02
	Village sanitation coverage						
	≤ 0.25	792	94.19	4.80	1.01	0.00	5.81
	0.26–0.54	1,123	94.57	5.08	0.36	0.00	5.43
	0.54–0.81	1,130	93.27	6.28	0.35	0.09	6.73
	> 0.81	1,059	94.24	5.38	0.38	0.00	5.76
***A*. *lumbricoides***	Overall	4,104	99.32	0.41	0.24	0.02	0.68

Hookworm intensity categories (epg = eggs per gram): none 0 epg; light <2,000 epg; moderate 2,000-<4,000 epg; heavy ≥4,000 epg

*T*. *trichiura* intensity categories: none 0 epg; light <1,000 epg; moderate 1,000-<10,000 epg; heavy ≥10,000 epg

*A*. *lumbricoides* intensity categories: none 0 epg; light <5,000 epg; moderate 5,000-<50,000 epg; heavy ≥50,000 epg

**Table 3:** Sanitation and contextual effects on presence of hookworm infection among 4,104 school-attending children in coastal Kenya.

	Intercept-Only		Crude		Adjusted^1^		
Fixed Effects	POR	(95% CI)	POR	(95% CI)	POR	(95% CI)	80% IOR
Household sanitation access	--	--	0.63	(0.51, 0.79)	0.76	(0.61, 0.95)	--
School sanitation coverage (per 100)							
1.49–2.17	--	--	0.89	(0.55, 1.42)	0.79	(0.51, 1.21)	(0.14, 4.52)
2.18–3.13	--	--	0.86	(0.54, 1.36)	0.64	(0.40, 0.98)	(0.11, 3.66)
> 3.13	--	--	0.58	(0.35, 0.98)	0.51	(0.31, 0.83)	(0.09, 2.94)
Village sanitation coverage							
0.26–0.54	--	--	1.28	(0.89, 1.86)	1.40	(0.96, 2.08)	(0.33, 6.06)
0.54–0.81	--	--	1.52	(1.01, 2.29)	1.86	(1.22, 2.86)	(0.43, 8.01)
> 0.81	--	--	1.21	(0.75, 1.92)	1.40	(0.86, 2.34)	(0.33, 6.06)
**Contextual Effects**							
**School**							
Variance	1.11	(0.92, 1.31)	1.12	(0.93, 1.32)	0.93	(0.74, 1.14)	--
MOR	2.73	(2.50, 2.98)	2.74	(2.51, 2.99)	2.51	(2.27, 2.77)	--
VPC	0.22	(0.20, 0.24)	0.22	(0.20, 0.24)	0.19	(0.17, 0.21)	--
**Village**							
Variance	0.60	(0.34, 0.83)	0.61	(0.34, 0.85)	0.65	(0.41, 0.89)	--
MOR	2.09	(1.74, 2.38)	2.11	(1.74, 2.41)	2.16	(1.84, 2.46)	--
VPC	0.12	(0.07, 0.15)	0.12	(0.07, 0.16)	0.13	(0.09, 0.17)	--

POR = Prevalence Odds Ratio; CI = Credible Interval; MOR = Median Odds Ratio; IOR = Interval Odds Ratio; VPC = Variance Partition Coefficient

^1^Adjusted for age (centred at 9), being female, reported deworming in past year, observed shoe-wearing, household floor covered, village high soil sand content, village aridity index (scaled 100x), village urban/periurban/rural, school high soil sand content, school aridity index (scaled 100x), school urban/periurban/rural

**Table 4:** Sanitation and contextual effects on presence of *T*. *trichiura* infection among 4,104 school-attending children in coastal Kenya.

	Intercept-Only		Crude		Adjusted^1^		
Fixed Effects	POR	(95% CI)	POR	(95% CI)	POR	(95% CI)	80% IOR
Household sanitation access	--	--	0.95	(0.66, 1.38)	1.00	(0.68, 1.46)	--
School sanitation coverage (per 100)							
1.49–2.17	--	--	0.75	(0.36, 1.51)	1.23	(0.70, 2.23)	(0.29, 5.26)
2.18–3.13	--	--	0.85	(0.42, 1.70)	1.07	(0.60, 1.93)	(0.25, 4.57)
> 3.13	--	--	0.81	(0.36, 1.73)	1.06	(0.56, 1.95)	(0.25, 4.53)
Village sanitation coverage							
0.26–0.54	--	--	1.00	(0.51, 1.92)	0.59	(0.30, 1.15)	(0.09, 4.05)
0.54–0.81	--	--	1.57	(0.79, 3.16)	0.82	(0.41, 1.62)	(0.12, 5.57)
> 0.81	--	--	0.84	(0.37, 1.90)	0.30	(0.14, 0.68)	(0.04, 2.07)
**Contextual Effects**							
**School**							
Variance	1.26	(0.84, 1.67)	1.34	(0.92, 1.78)	0.64	(0.11, 1.10)	--
MOR	2.92	(2.40, 3.43)	3.02	(2.50, 3.57)	2.14	(1.37, 2.72)	--
VPC	0.22	(0.17, 0.26)	0.23	(0.18, 0.27)	0.13	(0.03, 0.19)	--
**Village**							
Variance	1.15	(0.75, 1.56)	1.17	(0.77, 1.59)	1.12	(0.75, 1.51)	--
MOR	2.78	(2.28, 3.29)	2.81	(2.31, 3.33)	2.74	(2.28, 3.23)	--
VPC	0.20	(0.15, 0.24)	0.20	(0.15, 0.24)	0.22	(0.18, 0.26)	--

POR = Prevalence Odds Ratio; CI = Credible Interval; MOR = Median Odds Ratio; IOR = Interval Odds Ratio; VPC = Variance Partition Coefficient

^1^Adjusted for household SES category, village high soil sand content, village aridity index (scaled 100x), village urban/periurban/rural, school high soil sand content, school aridity index (scaled 100x), school urban/periurban/rural

In order to explore changes in these general contextual effects, inclusion of either a crude or a household sanitation measure adjusted for individual confounders in the model for hookworm infection did not change estimated MORs or VPCs (S3 Table), though school MOR and VPC were slightly reduced adjusting for village and school confounders. Further including school and village sanitation effects without adjustment for confounders at these levels did not change general contextual effects. Including a crude household sanitation measure, or with adjustment for household SES, in the model for *T*. *trichiura* did not change estimated school and village general contextual effects (S4 Table). School MOR and VPC were reduced with further adjustment for confounders in these domains. Including crude fixed effects for sanitation at the village and school level in separate models for hookworm and *T*. *trichiura* did not meaningfully change measures of variance and heterogeneity (S3 Table and S4 Table). Including all domain sanitation measures in the crude model did not change calculated MOR or VPC values for hookworm or *T*. *trichiura* relative to the intercept-only model (Tables 3 and 4). Adjusting for all potential confounders, residual heterogeneity between schools decreased for *T*. *trichiura* and, to a lesser extent, hookworm infection, but residual heterogeneity between villages was unchanged.

Reported presence of household sanitation access reduced odds of hookworm infection by 37%, compared to no household sanitation access (POR 0.63, 95% CI 0.51, 0.79), among children in villages and schools with similar sanitation conditions (Table 3). Adjusting for potential confounders, this association was attenuated towards null (POR 0.76, 95% CI 0.61, 0.95). School sanitation coverage in the two highest quartiles (> 2.17 toilets per 100 students) was associated with lower hookworm prevalence, compared to the lowest coverage quartile (< 1.49 toilets per 100 students), adjusting for potential confounders and household and village sanitation (POR 0.64, 95% CI 0.40, 0.98; POR 0.51, 95% CI 0.31, 0.83). No evidence of association of household or school sanitation with *T*. *trichiura* infection was detected (Table 4). Children in villages where 54 to 81% of households had sanitation access, compared to children in villages with ≤ 25% sanitation coverage, had 1.86 times the odds of hookworm infection (POR 1.86, 95% CI 1.22, 2.86). Children in villages where > 81% of households had sanitation access, compared to children in villages with ≤ 25% sanitation coverage, had 70% lower odds of *T*. *trichiura* infection (POR 0.30, 95% CI 0.14, 0.68). Results were robust to the exclusion of 199 children without household coordinates and 197 reporting attending a school > 6.5 km from their house.

Calculated 80% IORs from adjusted models for hookworm and *T*. *trichiura* were wide and included one for both village and school sanitation measures, indicating sanitation was less important for explaining individual infection, compared to residual variation between these domain levels. For example, comparing children with identical characteristics but drawn from either a village with sanitation coverage ≤ 25% or a village with sanitation coverage > 81%, odds of *T*. *trichiura* infection will be between 0.04 and 2.07 in 80% of such comparisons.

## Discussion

In this analysis of baseline data from the TUMIKIA trial, we found notable differences between two species of STH in the relationship between sanitation availability and prevalence of infection within different domains among school-attending children in coastal Kenya. Reported use of a sanitation facility by households was associated with reduced prevalence of hookworm infection but was not associated with reduced prevalence of *T*. *trichiura* infection. Meanwhile, village sanitation coverage > 81% was associated with reduced prevalence of *T*. *trichiura* infection, but no protective association was detected for hookworm infection. School sanitation coverage > 3.13 toilets per 100 pupils was associated with lower prevalence of hookworm infection. This coverage level, corresponding to a pupil:toilet ratio of 32:1, supports the minimum ratios (25:1 for girls and 35:1 for boys) currently recommended by the Kenyan government [32]. School sanitation was not associated with *T*. *trichiura* infection, however. We found that general contextual effects, represented by residual heterogeneity between village and school domains, had comparable impact upon likelihood of hookworm and *T*. *trichiura* infection as sanitation coverage in either of these domains.

Three published meta-analyses record considerable heterogeneity in estimates of the effect of sanitation access on hookworm and *T*. *trichiura* infection. Ziegelbauer *et al*. found that sanitation was protective against both hookworm and *T*. *trichiura* infection [5]. Strunz *et al*. found no association of sanitation access with hookworm infection, but it was protective against *T*. *trichiura* [4]. Freeman *et al*., in the most recent review, found no association of sanitation access with *T*. *trichiura* infection but sanitation access was protective against hookworm [6]. Our finding of association of household and school sanitation with hookworm infection is consistent with this latter result, while adjusting for village sanitation coverage plus potential confounders and conditional on village and school membership. Albendazole, the medication used for Kenya’s National School-Based Deworming Programme (NSBDP), is less effective against *T*. *trichiura*, compared to hookworm and *A*. *lumbricoides* [33]. The observed associations of household and school sanitation with lower hookworm prevalence in this population of school-attending children could reflect impacts of sanitation in these domains on reinfection, following school-based deworming. In contrast, due to albendazole’s lower effectiveness against *T*. *trichiura* infection, Freeman *et al*. concluded that only a long-term impact of sanitation access might be observable following deworming, which our finding would also support [6].

The three meta-analyses described above did not distinguish between sanitation access at home or at school. We estimated the independent effects of sanitation access at both the household and school on STH infection. We also expanded upon previous work to estimate the effect of village sanitation coverage [9, 10]. The protective association of village sanitation coverage against *T*. *trichiura* may reflect longer-term impacts of sanitation and a possible community-wide (herd) effect at access levels > 80%, independent of household sanitation access and other factors. In contrast, we found no consistent pattern between village sanitation coverage and hookworm infection. We may not have observed an association because many of the included villages with the lowest sanitation coverage were also in the most arid environments, limiting their suitability for transmission.

Our results clearly show the large contextual effects of village and school domains relative to the estimated fixed effects. The 80% IOR does not indicate precision but provides an interval around our estimated village and school sanitation effects that incorporates unexplained variability between these domains. This result, coupled with the calculated VPCs and MORs, indicates that sanitation coverage in these domains is not a strong predictor of hookworm or *T*. *trichiura* infection in this setting, though some protective associations were observed. Others have also reported that village membership alone has a large impact on the likelihood of hookworm or *T*. *trichiura* infection [34], and that heterogeneity of prevalence is associated with multiple environmental and socioeconomic factors [35]. Adjusting for potential individual, village and school level confounders in our models did not meaningfully explain further heterogeneity in hookworm infection between villages or schools but did explain some heterogeneity in *T*. *trichiura* infection between schools.

Though our general contextual effects indicate that village is a relevant context for analysis, the representativeness of village measures is a limitation of the current study. We aggregated village measures from all households sampled for the baseline survey. Because sampling for TUMIKIA was based on CUs, the number of units sampled per village varied. We assumed that included units were representative, but our sample may not have adequately characterised village conditions. Village membership was based on an administrative rather than geographic grouping, so it may also not represent sanitation conditions in the area surrounding study households. While useful for implementation purposes, village may not be the most suitable scale at which to assess community-wide sanitation effects. Future studies could use varying spatial buffers with complete household samples to examine community-wide effects of sanitation coverage on STH infection and identify target thresholds [36]. Our household sanitation measure was based on a reported measure and may not reflect actual consistent usage and faeces disposal by household members or faecal contamination levels in the area. Our outcome measure was based on a single stool sample, which may also have underestimated prevalence [37]. Our assumption that the probability of complete data is independent of the outcome after adjusting for included covariates may have been incorrect. Only a small proportion of subjects were excluded due to missing data on covariates and outcomes, so we would expect the magnitude of bias from this source to be minimal. We attempted to control for imbalance in characteristics and conditions between individuals in different exposure groups, but, as with any observational research study, potential residual confounding and our cross-sectional design remain important limitations.

In the current study, sanitation conditions, as measured, explained little of the heterogeneity in transmission between villages and schools. Further studies should examine the role of sanitation in different domains against STH infections within the context of school- and community-based mass drug administration (MDA). We found evidence of a protective effect of sanitation access at the household against hookworm infection and a sanitation coverage threshold at which a community-wide effect against *T*. *trichiura* was observed. We also found evidence in support of current school sanitation coverage guidelines towards the control of hookworm infection. In summary, our findings highlight the need for continued efforts, alongside MDA, to extend access to good sanitation facilities at homes, schools, and across communities.

## Supporting information

S1 Fig Sanitation and hookworm infection directed acyclic graph. (DOCX)

S2 Fig Sanitation and *Trichuris trichiura* infection directed acyclic graph. (DOCX)

S1 Text Sanitation and hookworm infection model code (dagitty.net). (DOCX)

S2 Text Sanitation and *Trichuris trichiura* infection model code (dagitty.net). (DOCX)

S1 Table STROBE checklist. (DOCX)

S2 Table Characteristics of school-attending children in non-arid areas by linked to school survey information status. (DOCX)

S3 Table Sanitation exposures and contextual effects on presence of hookworm infection among 4,104 school-attending children in coastal Kenya. (DOCX)

S4 Table Sanitation exposures and contextual effects on presence of *T. trichiura* infection among 4,104 school-attending children in coastal Kenya. (DOCX)
