# Supplementary figures and images for: Domains of transmission and association of community, school, and household sanitation with soil-transmitted helminth infections among children in coastal Kenya
Source: PLoS Negl Trop Dis. 2019 Nov 25;13(11):e0007488. doi: 10.1371/journal.pntd.0007488 (PMC6901232; doi:10.1371/journal.pntd.0007488)

**S1 Figure. Sanitation and hookworm infection directed acyclic graph**

**
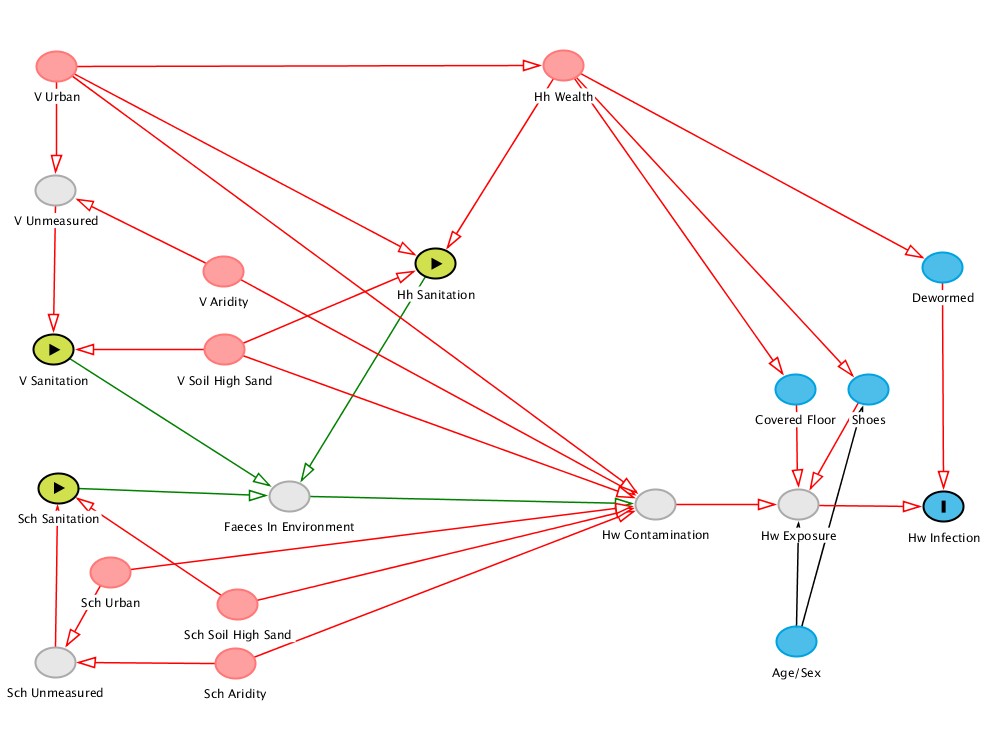
**

Supplement: S1 Fig — (DOCX) [file pntd.0007488.s001.docx]

**S2 Figure. Sanitation and *Trichuris trichiura* infection directed acyclic graph**

**
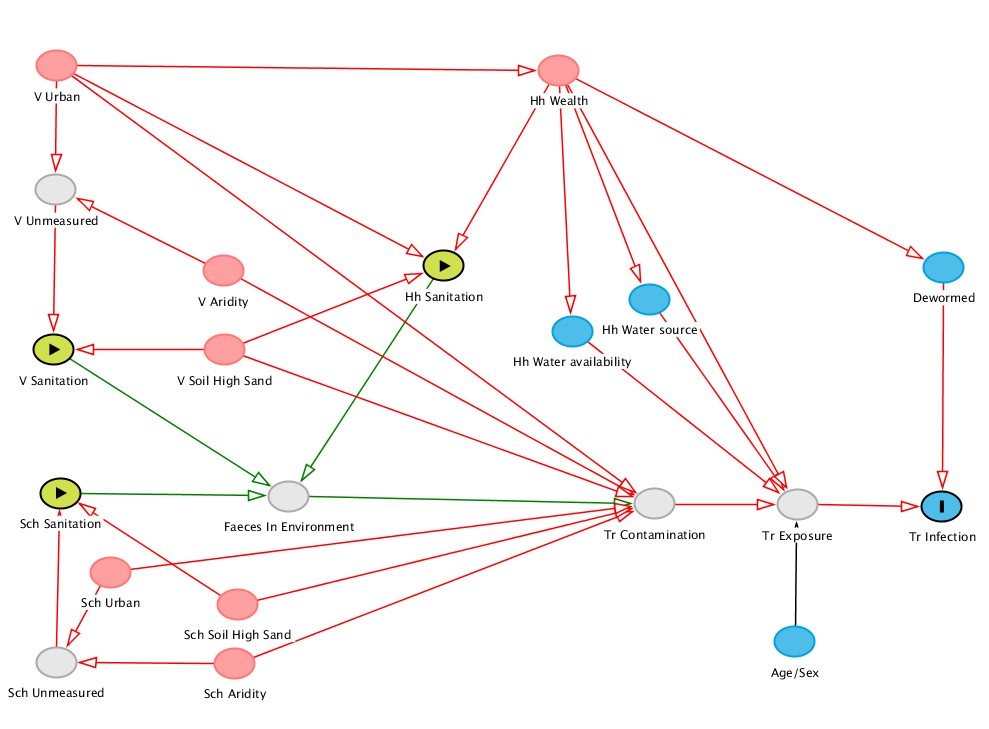
**

Supplement: S2 Fig — (DOCX) [file pntd.0007488.s002.docx]
