# Supplementary material for: Domains of transmission and association of community, school, and household sanitation with soil-transmitted helminth infections among children in coastal Kenya
Source: PLoS Negl Trop Dis. 2019 Nov 25;13(11):e0007488. doi: 10.1371/journal.pntd.0007488 (PMC6901232; doi:10.1371/journal.pntd.0007488)
Supplement: S1 Text — (DOCX) [file pntd.0007488.s003.docx]

**S1 Text. Sanitation and hookworm infection model code (dagitty.net)**

Age%2FSex 1 @0.753,1.348

Covered%20Floor 1 @0.751,0.156

Dewormed 1 @1.270,-0.420

Faeces%20In%20Environment U @-1.037,0.661

Hh%20Sanitation E @-0.521,-0.437

Hh%20Wealth 1 @-0.069,-1.372

Hw%20Contamination U @0.256,0.697

Hw%20Exposure U @0.761,0.701

Hw%20Infection O @1.273,0.709

Sch%20Aridity 1 @-1.228,1.449

Sch%20Sanitation E @-1.891,0.647

Sch%20Soil%20High%20Sand 1 @-1.220,1.170

Sch%20Unmeasured U @-1.898,1.435

Sch%20Urban 1 @-1.669,1.018

Shoes 1 @1.007,0.156

V%20Aridity 1 @-1.269,-0.402

V%20Sanitation E @-1.870,-0.031

V%20Soil%20High%20Sand 1 @-1.266,-0.031

V%20Unmeasured U @-1.861,-0.784

V%20Urban 1 @-1.859,-1.370

Age%2FSex Hw%20Exposure Shoes

Covered%20Floor Hw%20Exposure

Dewormed Hw%20Infection

Faeces%20In%20Environment Hw%20Contamination

Hh%20Sanitation Faeces%20In%20Environment

Hh%20Wealth Covered%20Floor Dewormed Hh%20Sanitation Shoes

Hw%20Contamination Hw%20Exposure

Hw%20Exposure Hw%20Infection

Sch%20Aridity Hw%20Contamination Sch%20Unmeasured

Sch%20Sanitation Faeces%20In%20Environment

Sch%20Soil%20High%20Sand Hw%20Contamination Sch%20Sanitation

Sch%20Unmeasured Sch%20Sanitation

Sch%20Urban Hw%20Contamination Sch%20Unmeasured

Shoes Hw%20Exposure

V%20Aridity Hw%20Contamination V%20Unmeasured

V%20Sanitation Faeces%20In%20Environment

V%20Soil%20High%20Sand Hh%20Sanitation Hw%20Contamination V%20Sanitation

V%20Unmeasured V%20Sanitation

V%20Urban Hh%20Sanitation Hh%20Wealth Hw%20Contamination V%20Unmeasured
