# Supplementary material for: Domains of transmission and association of community, school, and household sanitation with soil-transmitted helminth infections among children in coastal Kenya
Source: PLoS Negl Trop Dis. 2019 Nov 25;13(11):e0007488. doi: 10.1371/journal.pntd.0007488 (PMC6901232; doi:10.1371/journal.pntd.0007488)
Supplement: S2 Text — (DOCX) [file pntd.0007488.s004.docx]

**S2 Text. Sanitation and *Trichuris trichiura* infection model code (dagitty.net)**

Age%2FSex 1 @0.753,1.348

Dewormed 1 @1.279,-0.418

Faeces%20In%20Environment U @-1.037,0.661

Hh%20Sanitation E @-0.489,-0.425

Hh%20Water%20availability 1 @-0.035,-0.118

Hh%20Water%20source 1 @0.238,-0.268

Hh%20Wealth 1 @-0.083,-1.347

Sch%20Aridity 1 @-1.228,1.449

Sch%20Sanitation E @-1.891,0.647

Sch%20Soil%20High%20Sand 1 @-1.220,1.170

Sch%20Unmeasured U @-1.898,1.435

Sch%20Urban 1 @-1.669,1.018

Tr%20Contamination U @0.256,0.697

Tr%20Exposure U @0.761,0.701

Tr%20Infection O @1.273,0.709

V%20Aridity 1 @-1.269,-0.402

V%20Sanitation E @-1.870,-0.031

V%20Soil%20High%20Sand 1 @-1.266,-0.031

V%20Unmeasured U @-1.861,-0.784

V%20Urban 1 @-1.859,-1.370

Age%2FSex Tr%20Exposure

Dewormed Tr%20Infection

Faeces%20In%20Environment Tr%20Contamination

Hh%20Sanitation Faeces%20In%20Environment

Hh%20Water%20availability Tr%20Exposure

Hh%20Water%20source Tr%20Exposure

Hh%20Wealth Dewormed Hh%20Sanitation Hh%20Water%20availability Hh%20Water%20source Tr%20Exposure

Sch%20Aridity Sch%20Unmeasured Tr%20Contamination

Sch%20Sanitation Faeces%20In%20Environment

Sch%20Soil%20High%20Sand Sch%20Sanitation Tr%20Contamination

Sch%20Unmeasured Sch%20Sanitation

Sch%20Urban Sch%20Unmeasured Tr%20Contamination

Tr%20Contamination Tr%20Exposure

Tr%20Exposure Tr%20Infection

V%20Aridity Tr%20Contamination V%20Unmeasured

V%20Sanitation Faeces%20In%20Environment

V%20Soil%20High%20Sand Hh%20Sanitation Tr%20Contamination V%20Sanitation

V%20Unmeasured V%20Sanitation

V%20Urban Hh%20Sanitation Hh%20Wealth Tr%20Contamination V%20Unmeasured
