# Supplementary material for: Domains of transmission and association of community, school, and household sanitation with soil-transmitted helminth infections among children in coastal Kenya
Source: PLoS Negl Trop Dis. 2019 Nov 25;13(11):e0007488. doi: 10.1371/journal.pntd.0007488 (PMC6901232; doi:10.1371/journal.pntd.0007488)
Supplement: S2 Table — (DOCX) [file pntd.0007488.s006.docx]

**S2 Table. Characteristics of school-attending children in non-arid areas by linked to school survey information status**

|  | | **Not linked to school**  **N=613** | | **Linked to school**  **N=4,104** | |  |
| --- | --- | --- | --- | --- | --- | --- |
| **Characteristics** | | **No. or Mean** | **% or SD** | **No. or Mean** | **% or SD** | **p^1^** |
| **Individual level (n=4,104)** | |  |  |  |  |  |
|  | Hookworm infection | 120 | 19.58 | 730 | 17.79 | 0.283 |
|  | *T. trichiura* infection | 32 | 5.23 | 244 | 5.95 | 0.481 |
|  | Missing | 1 |  | 0 |  |  |
|  | Age (years) | 8.09 | 2.55 | 9.46 | 2.65 | <0.001 |
|  | Being girls | 290 | 47.31 | 2,129 | 51.88 | 0.035 |
|  | Observed wearing shoes | 207 | 33.88 | 1,443 | 35.16 | 0.535 |
|  | Missing | 2 |  | 0 |  |  |
|  | Reported deworming in past year | 199 | 33.11 | 2,219 | 54.07 | <0.001 |
|  | Missing | 12 |  | 0 |  |  |
|  | Reported household access to toilet | 358 | 58.50 | 2,255 | 54.95 | 0.099 |
|  | Missing | 1 |  | 0 |  |  |
|  | Reported improved water source | 344 | 56.12 | 2,216 | 54.17 | 0.366 |
|  | Missing | 0 |  | 13 |  |  |
|  | Time to fetch water, < 30 min | 516 | 84.45 | 3,344 | 81.76 | 0.105 |
|  | Missing | 2 |  | 14 |  |  |
|  | Covered floor | 172 | 28.06 | 833 | 20.30 | <0.001 |
|  | Household wealth quintile |  |  |  |  | <0.001 |
|  | Most poor, 1 | 148 | 24.14 | 1,190 | 29.00 |  |
|  | 2 | 314 | 51.22 | 2,158 | 52.58 |  |
|  | Least poor, 3 | 151 | 24.63 | 756 | 18.42 |  |

^1^Pearson chi-squared test or Two-sample t-test with unequal variances
