# Supplementary material for: Domains of transmission and association of community, school, and household sanitation with soil-transmitted helminth infections among children in coastal Kenya
Source: PLoS Negl Trop Dis. 2019 Nov 25;13(11):e0007488. doi: 10.1371/journal.pntd.0007488 (PMC6901232; doi:10.1371/journal.pntd.0007488)
Supplement: S3 Table — (DOCX) [file pntd.0007488.s007.docx]

**S3 Table. Sanitation exposures and contextual effects on presence of hookworm among 4,104 school-attending children in coastal Kenya**

|  | **Household Crude** | | **School Crude** | | **Village Crude** | | **Household, adjusted^1^** | | **Household, adjusted^2^** | | **Combined, adjusted^1^** | |
| --- | --- | --- | --- | --- | --- | --- | --- | --- | --- | --- | --- | --- |
| **Fixed Effects** | **POR** | **(95% CI)** | **POR** | **(95% CI)** | **POR** | **(95% CI)** | **POR** | **(95% CI)** | **POR** | **(95% CI)** | **POR** | **(95% CI)** |
|  |  |  |  |  |  |  |  |  |  |  |  |  |
| Household sanitation access | 0.65 | (0.53, 0.80) | -- | -- | -- | -- | 0.81 | (0.66, 1.00) | 0.80 | (0.65, 1.00) | 0.76 | (0.60, 0.96) |
|  |  |  |  |  |  |  |  |  |  |  |  |  |
| School sanitation coverage (per 100) |  |  |  |  |  |  |  |  |  |  |  |  |
| 1.49 - 2.17 | -- | -- | 0.90 | (0.57, 1.45) | -- | -- | -- | -- | -- | -- | 0.84 | (0.53, 1.35) |
| 2.18 - 3.13 | -- | -- | 0.84 | (0.52, 1.35) | -- | -- | -- | -- | -- | -- | 0.83 | (0.52, 1.31) |
| > 3.13 | -- | -- | 0.56 | (0.32, 0.95) | -- | -- | -- | -- | -- | -- | 0.61 | (0.36, 1.02) |
|  |  |  |  |  |  |  |  |  |  |  |  |  |
| Village sanitation coverage |  |  |  |  |  |  |  |  |  |  |  |  |
| 0.26 - 0.54 | -- | -- | -- | -- | 1.15 | (0.79, 1.70) | -- | -- | -- | -- | 1.36 | (0.94, 1.97) |
| 0.54 - 0.81 | -- | -- | -- | -- | 1.20 | (0.80, 1.80) | -- | -- | -- | -- | 1.67 | (1.11, 2.51) |
| > 0.81 | -- | -- | -- | -- | 0.82 | (0.53, 1.26) | -- | -- | -- | -- | 1.40 | (0.88, 2.27) |
|  |  |  |  |  |  |  |  |  |  |  |  |  |
| **Contextual Effects** |  |  |  |  |  |  |  |  |  |  |  |  |
| **School** |  |  |  |  |  |  |  |  |  |  |  |  |
| Variance | 1.12 | (0.93, 1.33) | 1.13 | (0.94, 1.33) | 1.09 | (0.90, 1.29) | 1.09 | (0.90, 1.30) | 0.97 | (0.78, 1.18) | 1.09 | (0.91, 1.30) |
| MOR | 2.74 | (2.51, 3.00) | 2.76 | (2.52, 3.00) | 2.71 | (2.47, 2.95) | 2.71 | (2.47, 2.97) | 2.56 | (2.32, 2.82) | 2.71 | (2.48, 2.97) |
| VPC | 0.22 | (0.20, 0.24) | 0.23 | (0.21, 0.24) | 0.22 | (0.20, 0.24) | 0.22 | (0.20, 0.24) | 0.20 | (0.17, 0.22) | 0.22 | (0.20, 0.24) |
|  |  |  |  |  |  |  |  |  |  |  |  |  |
| **Village** |  |  |  |  |  |  |  |  |  |  |  |  |
| Variance | 0.60 | (0.35, 0.83) | 0.59 | (0.33, 0.82) | 0.62 | (0.38, 0.84) | 0.61 | (0.35, 0.85) | 0.66 | (0.40, 0.90) | 0.60 | (0.34, 0.85) |
| MOR | 2.09 | (1.76, 2.38) | 2.08 | (1.73, 2.37) | 2.12 | (1.80, 2.40) | 2.11 | (1.76, 2.41) | 2.16 | (1.83, 2.47) | 2.09 | (1.74, 2.41) |
| VPC | 0.12 | (0.08, 0.15) | 0.12 | (0.07, 0.15) | 0.12 | (0.08, 0.15) | 0.12 | (0.08, 0.16) | 0.13 | (0.09, 0.17) | 0.12 | (0.07, 0.16) |
|  |  |  |  |  |  |  |  |  |  |  |  |  |

POR = Prevalence Odds Ratio; CI = Credible Interval; MOR = Median Odds Ratio; VPC = Variance Partition Coefficient

^1^Adjusted for age (centred at 9), being female, reported deworming in past year, observed shoe-wearing, household floor covered

^2^Adjusted for age (centred at 9), being female, reported deworming in past year, observed shoe-wearing, household floor covered, village high soil sand content, village aridity index (scaled 100x), village urban/periurban/rural, school high soil sand content, school aridity index (scaled 100x), school urban/periurban/rural
