# Supplementary material for: Domains of transmission and association of community, school, and household sanitation with soil-transmitted helminth infections among children in coastal Kenya
Source: PLoS Negl Trop Dis. 2019 Nov 25;13(11):e0007488. doi: 10.1371/journal.pntd.0007488 (PMC6901232; doi:10.1371/journal.pntd.0007488)
Supplement: S4 Table — (DOCX) [file pntd.0007488.s008.docx]

**S4 Table. Sanitation exposures and contextual effects on presence of *T. trichiura* among 4,104 school-attending children in coastal Kenya**

|  | **Household Crude** | | **School Crude** | | **Village Crude** | | **Household, adjusted^1^** | | **Household, adjusted^2^** | | **Combined, adjusted^1^** | |
| --- | --- | --- | --- | --- | --- | --- | --- | --- | --- | --- | --- | --- |
| **Fixed Effects** | **POR** | **(95% CI)** | **POR** | **(95% CI)** | **POR** | **(95% CI)** | **POR** | **(95% CI)** | **POR** | **(95% CI)** | **POR** | **(95% CI)** |
|  |  |  |  |  |  |  |  |  |  |  |  |  |
| Household sanitation access | 0.96 | (0.68, 1.34) | -- | -- | -- | -- | 1.02 | (0.73, 1.43) | 0.85 | (0.60, 1.21) | 1.02 | (0.70, 1.48) |
|  |  |  |  |  |  |  |  |  |  |  |  |  |
| School sanitation coverage (per 100) |  |  |  |  |  |  |  |  |  |  |  |  |
| 1.49 - 2.17 | -- | -- | 0.76 | (0.38, 1.58) | -- | -- | -- | -- | -- | -- | 0.76 | (0.37, 1.54) |
| 2.18 - 3.13 | -- | -- | 0.83 | (0.41, 1.67) | -- | -- | -- | -- | -- | -- | 0.86 | (0.43, 1.75) |
| > 3.13 | -- | -- | 0.79 | (0.36, 1.72) | -- | -- | -- | -- | -- | -- | 0.84 | (0.38, 1.84) |
|  |  |  |  |  |  |  |  |  |  |  |  |  |
| Village sanitation coverage |  |  |  |  |  |  |  |  |  |  |  |  |
| 0.26 - 0.54 | -- | -- | -- | -- | 0.99 | (0.51, 1.93) | -- | -- | -- | -- | 0.99 | (0.50, 1.97) |
| 0.54 - 0.81 | -- | -- | -- | -- | 1.54 | (0.79, 3.03) | -- | -- | -- | -- | 1.55 | (0.77, 3.16) |
| > 0.81 | -- | -- | -- | -- | 0.82 | (0.38, 1.70) | -- | -- | -- | -- | 0.84 | (0.38, 1.84) |
|  |  |  |  |  |  |  |  |  |  |  |  |  |
| **Contextual Effects** |  |  |  |  |  |  |  |  |  |  |  |  |
| **School** |  |  |  |  |  |  |  |  |  |  |  |  |
| Variance | 1.27 | (0.85, 1.69) | 1.31 | (0.90, 1.73) | 1.32 | (0.90, 1.75) | 1.28 | (0.85, 1.72) | 0.66 | (0.14, 1.09) | 1.35 | (0.91, 1.80) |
| MOR | 2.93 | (2.41, 3.46) | 2.98 | (2.47, 3.51) | 2.99 | (2.47, 3.53) | 2.94 | (2.41, 3.49) | 2.17 | (1.43, 2.71) | 3.03 | (2.48, 3.60) |
| VPC | 0.22 | (0.17, 0.26) | 0.23 | (0.18, 0.26) | 0.23 | (0.18, 0.27) | 0.22 | (0.17, 0.26) | 0.13 | (0.03, 0.19) | 0.23 | (0.18, 0.27) |
|  |  |  |  |  |  |  |  |  |  |  |  |  |
| **Village** |  |  |  |  |  |  |  |  |  |  |  |  |
| Variance | 1.16 | (0.77, 1.58) | 1.16 | (0.77, 1.58) | 1.15 | (0.75, 1.56) | 1.18 | (0.79, 1.59) | 1.08 | (0.71, 1.47) | 1.18 | (0.78, 1.60) |
| MOR | 2.79 | (2.31, 3.32) | 2.79 | (2.31, 3.32) | 2.78 | (2.28, 3.29) | 2.82 | (2.33, 3.33) | 2.69 | (2.23, 3.18) | 2.82 | (2.32, 3.34) |
| VPC | 0.20 | (0.16, 0.24) | 0.20 | (0.16, 0.24) | 0.20 | (0.15, 0.24) | 0.21 | (0.16, 0.24) | 0.21 | (0.17, 0.25) | 0.20 | (0.16, 0.24) |
|  |  |  |  |  |  |  |  |  |  |  |  |  |

POR = Prevalence Odds Ratio; CI = Credible Interval; MOR = Median Odds Ratio; VPC = Variance Partition Coefficient

^1^Adjusted for household SES category

^2^ Adjusted for household SES category, village high soil sand content, village aridity index (scaled 100x), village urban/periurban/rural, school high soil sand content, school aridity index (scaled 100x), school urban/periurban/rural
